# Association of *THBS1* genetic variants and mRNA expression with the risks of ischemic stroke and long-term death after stroke

**DOI:** 10.3389/fnagi.2022.1006473

**Published:** 2022-09-23

**Authors:** Changying Chen, Xuemei Chen, Siyuan Yang, Qingqing Li, Zhanyun Ren, Lu Wang, Yuzhang Jiang, Xincheng Gu, Fangyuan Liu, Jialing Mu, Lihua Liu, Yi Wang, Junrong Li, Yanhua Yu, Jun Zhang, Chong Shen

**Affiliations:** ^1^Department of Epidemiology, School of Public Health, Nanjing Medical University, Nanjing, China; ^2^Department of Neurology, The Affiliated Jiangning Hospital of Nanjing Medical University, Nanjing, China; ^3^Department of Neurology, The Affiliated Hospital of Xuzhou Medical University, Xuzhou, China; ^4^Department of Neurology, The Affiliated Yixing Hospital of Jiangsu University, Yixing, China; ^5^Department of Neurology, Jurong Hospital Affiliated to Jiangsu University, Jurong People’s Hospital, Jurong, China; ^6^Department of Medical Laboratory, Huai’an First People’s Hospital, The Affiliated Huai’an No.1 People’s Hospital of Nanjing Medical University, Huai’an, China; ^7^Suzhou Center for Disease Control and Prevention, Suzhou, China

**Keywords:** thrombospondin-1, mRNA expression, ischemic stroke, case control study, cohort study

## Abstract

**Background:**

Thrombospondin-1 (THBS1) derived from platelets and acted as a critical mediator of hemostasis promoting platelet activation in thrombus formation. The biological connection of genetic variants and mRNA expression of *THBS1* with ischemic stroke (IS) warrants further validation with population-based evidence.

**Objective:**

To evaluate the association of single nucleotide polymorphisms (SNPs) and mRNA expression of *THBS1* with the risks of IS and long-term death after stroke.

**Methods:**

A case-control study consisted of 4,584 IS patients recruited from five hospitals in Jiangsu, China, and 4,663 age-gender-matched controls free of IS. A cohort study enrolled 4,098 participants free of stroke and lasted from 2009 to 2022. Early collected 3158 IS patients aged between 35 and 80 years were followed up an average of 5.86-year to follow up their long-term death outcomes. Two tagSNPs of the *THBS1* gene, rs2236471 and rs3743125, were genotyped in all subjects and *THBS1* mRNA expression of peripheral leukocyte was measured using RT-qPCR in 314 IS cases and 314 controls.

**Results:**

There is no significant difference in genotype and haplotype frequencies of rs2236741 and rs3743125 between IS cases and controls (all *P* > 0.05). Furthermore, the cohort studies did not observe significant associations between *THBS1* variants and the risk of IS incidence or long-term death after IS (all *P* > 0.05). The *THBS1* mRNA expression level (2^–Δ^ ^Δ^ *^CT^*) in IS cases was approximately equal to that in controls (1.01 vs. 0.99, *P* = 0.833). In addition, *THBS1* mRNA expression had no significant association with all-cause death, stroke death, and IS death of IS patients (all *P* > 0.05).

**Conclusion:**

Therefore, our study suggested that there is no significant association of *THBS1* polymorphisms and mRNA expression level with the risk of IS and long-term death after IS.

## Introduction

Stroke affects 13.7 million people worldwide every year and it is the second leading cause of death, with 5.5 million people dying from it annually (Feigin et al., 2018). The main subtypes of stroke include ischemic stroke (IS) and hemorrhagic stroke (HS). IS, making up ∼71% of all strokes, was defined as infarction of the brain, spinal cord, or retina (Virani et al., 2021). As the 2020 report on cardiovascular health and diseases burden in China, the number of patients suffering from stroke in China was about 13 million at present and the deaths of stroke among Chinese residents accounted for 22.33% of the total deaths (Xiao et al., 2021). Thus, exploring the cause of stroke is important as it can guide therapeutic strategies for the prevention of stroke.

Previous studies presented that a history of hypertension or diabetes mellitus, high levels of blood pressure, smoking, high alcohol consumption were considered as the modifiable risk factors for IS (O’Donnell et al., 2010), while age, gender, and genetic factors are non-modifiable risk factors (Campbell et al., 2019). The estimated heritability of IS was about 37.9% when calculated by current genome-wide complex trait analysis (Bevan et al., 2012). Therefore, the missing heritability of IS and gene-environment interaction remain further exploration.

Thrombospondin-1 (THBS1) is a multifunctional glycoprotein released from platelets, macrophages, and adipocytes (Baenziger et al., 1972; Jaffe et al., 1985; Varma et al., 2008). Endogenous THBS1 is necessary for platelet aggregation and adhesion by overcoming the antithrombotic activity of physiologic nitric oxid (NO) (Isenberg et al., 2008). Besides, THBS1 is a critical mediator of hemostasis that promotes platelet activation by modulating inhibitory cyclic adenosine monophosphate (cAMP) signaling at sites of vascular injury (Aburima et al., 2021). Additionally, THBS1 participates in a wide range of physiological and pathological processes such as tissue remodeling, wound healing, angiogenesis, and inflammation (Sweetwyne and Murphy-Ullrich, 2012). THBS1 is also known to regulate the activation of transforming growth factor-β1 (*TGF-*β*1*) (Sweetwyne and Murphy-Ullrich, 2012), which makes a difference in restenosis after angioplasty, atherosclerosis, and angiogenesis (August and Suthanthiran, 2006). Above all functions may be involved in the pathophysiology of IS, with common sources of embolism being large artery atherosclerosis (Campbell et al., 2019).

Previous studies showed that genetic variants in the *THBS1* gene were associated with variation in pulmonary artery systolic pressure (Jacob et al., 2017), and chronic ocular surface inflammation after refractive surgery (Contreras-Ruiz et al., 2014). Besides, the coding polymorphism G1678A (rs2292305) was identified as a susceptible locus for cerebral thrombosis in a Chinese population (Liu et al., 2004) and the *THBS1* CT genotype was associated with a reduced risk of developing gastric cancer (Hong et al., 2015). Furthermore, the visceral *THBS1* mRNA expression was positively associated with abdominal obesity, hyperglycemia, and hypertension (Matsuo et al., 2015). Additionally, plasma THBS1 level elevated in patients with IS compared to the controls (Gao et al., 2015) as well as at 2h after tPA-treatment (Navarro-Sobrino et al., 2011). Thus, the serum THBS1 level was proposed to be an independent predictor of favorable outcome at baseline and after 6 months (Gao et al., 2015; Al Qawasmeh et al., 2020). Therefore, further population-based studies would be warranted to validate the association between *THBS1* and IS.

Therefore, this research aimed to investigate whether the genetic variants and mRNA expression of *THBS1* was associated with the susceptibility to developing IS and the long-term death after stroke by conducting the case-control and cohort studies in the Chinese population.

## Materials and methods

### Population in the case-control study

A total of 4,584 IS cases were recruited from five different hospitals in Jiangsu province, China, which included Jurong People’s Hospital, Nanjing Jiangning Hospital, Yixing People’s Hospital, the Affiliated Hospital of Xuzhou Medical University, and Huai’an First People’s Hospital from 2009 to 2021. Included patients were diagnosed according to the computed tomography (CT), magnetic resonance imaging (MRI), or angiography with symptoms lasting more than 24 h. Excluded patients were those who had non-atherosclerotic ischemic stroke, acute coronary syndrome, tumor, autoimmune disease, or severe kidney failure according to the patient’s history of disease, admission diagnosis and discharge diagnosis. Besides, we use the TOAST (Trial of ORG 10172 in Acute Stroke Treatment) criteria to group the IS subjects. The TOAST classification denotes five subtypes of ischemic stroke: large-artery atherosclerosis (LAA), cardioembolism (CE), small-vessel occlusion (SVO), stroke of other determined etiology (SOE), and stroke of undetermined etiology (SUE). 4,663 controls free of stroke were randomly sampled from local communities and matched to the cases on age (±2 years) and gender. Subjects with acute coronary syndrome, tumor, autoimmune disease, and severe kidney failure were also excluded from the study. Additionally, we chose 314 pairs of IS cases and controls to detect *THBS1* mRNA expression from 2019 to 2021. IS cases had a definite TOAST type and qualified retention samples. The controls were selected from the cohorts in the same or adjacent areas of IS cases using age- and gender- matching method. For above 314 IS patients, we collected the National Institutes of Health Stroke Scale (NIHSS) score and modified Rankin scale (mRS) score of IS patients when discharge, and followed-up their NIHSS scores and mRS scores at 1, 3, and 6 months after discharge. Demographic and clinical characteristics of the study subjects in above two parts were listed in Table 1.

**TABLE 1:** Demographic and clinical characteristics of the study population.

		Case-control study								
Characteristics	Group	Total population				Subgroups for mRNA comparison				Cohort study (*n* = 4098)
		Control (*n* = 4663)	IS (*n* = 4584)	*Z*/χ ^2^	*P*	Control (*n* = 314)	IS (*n* = 314)	*Z*/χ ^2^	*P*	
Age (year)		66 (60, 72)	66 (59, 72)	0.924	0.355^a^	66 (56, 73)	68 (57, 74)	1.386	0.166^a^	59 (52, 67)
Gender [*n* (%)]	Male	1941 (41.6)	2722 (59.4)	291.499	<0.001^b^	182 (58.0)	182 (58.0)	<0.001	1.000^b^	1663 (40.6)
	Female	2722 (58.4)	1862 (40.6)			132 (42.0)	132 (42.0)			2435 (59.4)
SBP (mmHg)		139 (127, 152)	150 (134, 161)	20.050	<0.001^a^	148 (133, 158)	154 (138, 168)	3.288	0.001^a^	134 (123, 141)
DBP (mmHg)		81 (74, 88)	87 (80, 95)	23.086	<0.001^a^	84 (76, 92)	85 (76, 95)	0.769	0.442^a^	82 (78, 89)
GLU (mmol/L)		5.45 (4.97, 5.97)	5.35 (4.56, 5.63)	16.924	<0.001^a^	5.86 (5.27, 6.76)	5.48 (4.88, 6.52)	3.875	<0.001^a^	5.28 (4.85, 5.80)
TC (mmol/L)		4.93 (4.32, 5.60)	4.52 (3.83, 5.28)	19.287	<0.001^a^	4.77 (4.21, 5.53)	4.45 (3.67, 5.15)	5.116	<0.001^a^	4.80 (4.22, 5.45)
TG (mmol/L)		1.36 (0.96, 1.96)	1.38 (0.98, 2.04)	1.822	0.068^a^	1.34 (0.92, 1.90)	1.28 (0.99, 1.88)	0.054	0.957^a^	1.32 (0.90, 2.00)
HDL-C (mmol/L)		1.32 (1.14, 1.54)	1.14 (0.97, 1.34)	28.142	<0.001^a^	1.31 (1.09, 1.56)	1.09 (0.93, 1.30)	8.119	<0.001^a^	1.33 (1.13, 1.55)
LDL-C (mmol/L)		2.71 (2.21, 3.21)	2.64 (2.09, 3.19)	3.724	<0.001^a^	2.73 (2.207, 3.24)	2.71 (2.03, 3.25)	0.702	0.483^a^	2.65 (2.20, 3.11)
Smoking [*n* (%)]	No	3725 (79.9)	3570 (77.9)	5.578	0.018^b^	232 (73.9)	266 (84.7)	11.214	0.001^b^	3103 (75.7)
	Yes	938 (20.1)	1014 (22.1)			82 (26.1)	48 (15.3)			995 (24.3)
Drinking [*n* (%)]	No	3748 (80.4)	3968 (86.6)	63.996	<0.001^b^	220 (70.1)	283 (90.1)	39.643	<0.001^b^	3215 (78.5)
	Yes	915 (19.6)	616 (13.4)			94 (29.9)	31 (9.9)			883 (21.5)
Hypertension [*n* (%)]	No	2327 (49.9)	734 (16.0)	1198.984	<0.001^b^	85 (27.1)	42 (13.4)	18.250	<0.001^b^	2110 (51.5)
	Yes	2336 (50.1)	3850 (84.0)			229 (72.9)	272 (86.6)			1988 (48.5)
Diabetes [*n* (%)]	No	4000 (85.8)	3327 (72.6)	244.928	<0.001^b^	236 (75.2)	208 (66.2)	6.027	0.014^b^	3634 (88.7)
	Yes	663 (14.2)	1257 (27.4)			78 (24.8)	106 (33.8)			464 (11.3)
Dyslipidemia [*n* (%)]	No	2891 (62.0)	2693 (58.7)	10.213	0.001^b^	152 (48.4)	241 (76.8)	53.862	<0.001^b^	1642 (40.1)
	Yes	1772 (38.0)	1891 (41.3)			162 (51.6)	73 (23.2)			2456 (59.9)

^a^Mann-Whitney *U*-test; ^b^χ^2^-test; IS, ischemic stroke; SBP, systolic blood pressure; DBP, diastolic blood pressure; GLU, glucose; TC, total cholesterol; TG, triglyceride; HDL-C, high-density lipoprotein-cholesterol; LDL-C, low-density lipoprotein-cholesterol.

### Population and outcome in the cohort study

This research incorporated two prospective cohort studies. The community-based cohort study was conducted from 2009 to 2022, which recruited 4128 participants from Guanlin Town and Xushe Town, Yixing city (Jiangsu, China). 4098 baseline subjects without stroke were followed up until May 25, 2022 for stroke onset. The detailed information about this cohort has been described previously (Dong et al., 2021).

The hospital-based cohort study enrolled 3158 IS patients aged between 35 and 80 years from Yixing People’s Hospital and followed an average of 5.86-year to record their long-term death outcome based on the annual death data from Yixing Center for Disease Control and Prevention. The follow-up period ended on May 25, 2022. The four endpoints were all-cause death, stroke death, IS death, and HS death. Finally, we observed a total of 488 deaths, among which 245 died from stroke and over half of the stroke-induced deaths were attributed to IS (*n* = 161). Meanwhile, we collected outcome of the long-term death from local Centers for Disease Control and Prevention (CDC) to assessment the prognosis of IS patients comprehensively, including all-cause death, stroke death, and IS death. Followed up to May 13, 2022, we observed a total of 28 deaths. Of the 20 deaths from stroke, 13 deaths were attributed to IS.

Demographic and clinical characteristics of the study population in the two cohort studies were summarized in Supplementary Table 2.

The case-control and cohort studies were all approved by the Research Ethics Committee of Nanjing Medical University (#2018571) and all participants signed the informed consent voluntarily.

### Questionnaire survey and physical examination

Demographic characteristics, smoking and drinking habits, diseases history, and medication history were collected by questionnaire survey. All investigators were trained and qualified uniformly. Subjects who have ever smoked one pack of cigarettes a day in the past were considered smokers. Drinker was defined as individuals drinking alcohol at least three times daily. Physical examination including systolic blood pressure (SBP, mmHg) and diastolic blood pressure (DBP, mmHg) were measured at least three times.

Hypertension was defined as a self-reported history of hypertension, or elevated levels of blood pressure (SBP ≥ 140 mmHg, DBP ≥ 90 mmHg), or taking antihypertensive drugs recently. Those with fasting blood glucose level (GLU) ≥ 7.0 mmol/L, or self-reported history of diabetes, or taking hypoglycemic drugs were considered as diabetes. Dyslipidemia was defined as abnormal changes in lipid levels [total cholesterol (TC) ≥ 6.2 mmol/L, triglyceride (TG) ≥ 2.3 mmol/L, low-density lipoprotein-cholesterol (LDL-C) ≥ 4.1 mmol/L, high-density lipoprotein-cholesterol (HDL-C) < 1.04 mmol/L], or self-reported diagnosis of dyslipidemia, or currently taking lipid-lowering drugs.

### Blood sample collection and biochemical index detection

Venous peripheral blood was collected from each subject after 8 h from the last meal into vacuum anticoagulation tubes with ethylene diamine tetraacetic acid dipotassium salt (EDTA-K2) and stored at –20°C. Leukocytes were obtained by gradient centrifugation from a subgroup of the study population. GLU was measured using the glucose oxidase method. TC was detected by the cholesterol oxidase-peroxidase method. TG was detected by the glycerophosphate oxidase-peroxidase method. HDL-C was detected by the catalase removal method and LDL-C was measured by sulfuric acid precipitation method.

### Single nucleotide polymorphism selection and genotyping

The *THBS1* gene (Gene ID: 7057, Locus NC_000015.10) locates on chromosome 15q14 and spans 18,388 bp, and consists of 22 exons. We searched the SNPs from the upstream 2 kb to the downstream 1 kb and selected tagging SNPs (tagSNPs) through the database of the Chinese Han population in Beijing (CHB) and China of the International Hap MAP Project. Three tagSNPs (tagSNPs rs2292305, etc. tagSNP rs2236741, and tagSNP rs2292304) would be available for candidate SNP selection. Included tagSNPs met the criteria of minor allele frequency (MAF) ≥ 0.05 and linkage disequilibrium (LD) *r*^2^ ≥ 0.8. A functional candidate strategy was also applied to select potential functional SNPs on the bioinformatics effect prediction website SNPinfo Web Server.^1^ The SNP function prediction results of rs2292305 showed no informed predictive biological function, therefore, we selected the closely linked tagSNP rs3743125 (*r*^2^ = 0.932) with a prior predictive biological function as the substitute. We did not include rs2292304 because its probes and primers were not designed successfully. Finally, two tagSNPs of *THBS1* gene, rs2236471 (C > T) and rs3743125 (G > A) were selected and genotyped in this study. Detailed biological information and function prediction were summarized in Supplementary Table 1.

DNA was extracted from the unfrozen venous peripheral blood using the protein precipitation method (Eaglink, EGEN2024, China) and then preserved at –20°C. Each DNA sample was quantified using the NanoDrop 2000 spectrophotometer (Thermo Fisher Scientific, Waltham, MA). The polymerase chain reaction (PCR) TaqMan MGB probe assay was performed to amplify the two SNPs in the GeneAmp^®^ PCR system 9700 thermal cycle (Applied Biosystems, Foster City, CA). The results were post-read on the 7900HT real-time PCR system (Applied Biosystems, Foster City, CA) with the Sequence Detection System (SDS) 2.4 software. The successful call rates of two SNPs genotyping were both 100%.

### RNA extraction, reverse transcription, and quantitative real-time polymerase chain reaction

White blood cells were separated from peripheral blood by gradient centrifugation and stored at –20°C. Peripheral leukocyte was reserved in RNA protective additive (Eaglink, EGEN2026, China) at –20°C. Total RNA was extracted using the Whole Blood RNA Extraction Kit (Yuan, Yu-BR02-1, China) and quantified using a NanoDrop 2000 spectrophotometer (Thermo Fisher Scientific, Waltham, MA). Isolated RNA was reversely transcribed into cDNA using the PrimeScript™ RT Reagent Kit (Takara, RR047A, Japan). *THBS1* gene mRNA expression of peripheral leukocyte was measured using SYBR Green quantitative real-time polymerase chain reaction (RT-qPCR), and the housekeeping gene Glyceraldehyde-3-Phosphate Dehydrogenase (*GAPDH*) was tested as an endogenous control. The qPCR reaction system included Platinum^®^ SYBR^®^ Green qPCR SuperMix-UDG (Invitrogen, 11733-046, USA), RNase free dH_2_O, forward primer, reverse primer, and cDNA. Samples were incubated at 95°C for 5 min, followed by 40 cycles of 95°C for 10 s, 57°C for 20 s, and 72°C for 20 s on the QuantStudio*™* 7 Flex Real-Time PCR System platform (Applied Biosystems, 4485700, CA). All samples were analyzed in three parallels, and cycle threshold values were recorded. The 2^–ΔΔCT^ method was used to calculate relative expression levels of *THBS1* normalized by *GAPDH*. *THBS1* mRNA’s forward primer sequence (5′–3′) was AGACTCCGCATCGCAAAGG, and the reverse primer sequence (5′–3′) was TCACCACGTTGTTGTCAAGGG. *GAPDH* mRNA’s forward primer sequence (5′–3′) was GGAGCGAGATCCCTCCAAAAT, and the reverse primer sequence (5′–3′) was GGCTGTTGTCATACTTCTCATGG.

### Statistical analysis

We used EpiData 3.1 software (The EpiData Association, Odense, Denmark) for duplicate entry and consistency check of the collected data. Continuous variables were presented as median [inter-quartile range (IQR)] for non-parametric data. Categorical variables were presented as frequencies and percentages. For group-wise comparisons, the Kruskal–Wallis test or Mann–Whitney test was used for continuous variables with abnormal distribution. The chi-square test (χ^2^) was used to compare the differences in categorical variables between case and control groups. The Fisher’s exact test was used to estimate whether the genotype frequencies in controls and IS group met the Hardy–Weinberg equilibrium (HWE) law. Binary logistic regression was applied to calculate the odds ratios (*OR*s) and corresponding 95% confidence intervals (*CI*s) for the association of *THBS1* variants and IS with adjustment for covariates (age, gender, smoking, drinking, hypertension, diabetes, and dyslipidemia). We used Cox proportional hazard regression to estimate the association with hazard ratios (*HR*s) and 95% *CI*s as well as after adjustment for covariates in the cohort study. Kruskal-Wallis H test was conducted to test the trend in mRNA levels among different groups of SNP genotypes. We used Spearman’s rank correlation to evaluate the correlations between *THBS1* mRNA expression and NIHSS scores and MRS Scores after discharge in IS cases. We also used restricted cubic splines (RCS) with four knots at the 20th, 40th, 60th, and 80th centiles to flexibly model the association of *THBS1* mRNA expression with the risk of long-term deaths in IS patients.

Haplotype association analyses as outlined by Schaid et al. (2002) were performed to test the associations of statistically inferred Haplotype with IS weighted with their estimated probability. All data analyses were carried out using SAS software 9.4 (SAS Inc., Cary, N.C., USA) and R 4.1.1 version.^2^ A two−tailed *P-*value < 0.05 was considered statistically significant.

## Results

### Demographic and clinical characteristics of the study population

Table 1 shows the detailed demographic and clinical characteristics of 4,584 IS cases and 4,663 matched controls in the genetic case-control study. The median age of IS group (66 years) was comparable with that of the control group (66 years, *P* = 0.355). IS group had higher proportions of male (59.4%) and smokers (22.1%) while a lower proportion of drinkers (13.4%) than control group (41.6, 20.1, and 19.6%; *P* < 0.05). There were significant differences in SBP, DBP, GLU, TC, HDL-C, and LDL-C levels (*P* < 0.001) between the IS cases and controls. IS group had higher prevalence of hypertension (84.0%), diabetes (27.4%), and dyslipidemia (41.3%) than controls (50.1, 14.2 and 38.0%; *P* ≤ 0.001).

In the case-control study for transcriptome level analysis, IS group presented higher level of SBP but lower levels of GLU, TC, and HDL-C than control group (Table 1). Age and gender were both matched for case and control. IS group had lower proportions of smokers (15.3%) and drinkers (9.9%) than control group (26.1% and 29.9%; *P* ≤ 0.001). IS group had higher prevalence of hypertension (86.6%) while a lower prevalence of dyslipidemia (23.2%) than control group (72.9 and 51.6%; *P* < 0.001). Table 1 also listed the characteristics of participants in the cohort study for the risk of IS incidence. The clinical characteristic of the follow-up study for the long-term death after IS were presented in Supplementary Table 2.

### Association analyses of the thrombospondin-1 variants with IS in the case-control study

As shown in Table 2, the genotype and allele distributions of SNP rs2236741 followed *HWE* both in the control and IS groups (both *P-*values > 0.05). Even though we have double-checked the genotyping results and controlled the quality by comparing the genotype frequencies of cases and controls of each plate, the allele frequencies of rs3743125 did not accord with the *HWE* in controls (*P* = 0.024) but accord with *HWE* in IS cases (*P* = 0.098).

**TABLE 2:** Association analyses of *THBS1* variants and the risk of ischemic stroke in the case-control study.

SNP	Group	WT/HT/MT	*OR* (95% *CI*)^a^			Allele			*P* for *HWE*
			Additive model	Dominant model	Recessive model	Major/Minor	*OR* (95% *CI*)	*P* ^b^	
rs2236741	Control	3445/1116/102	0.955 (0.872–1.047)	0.973 (0.877–1.078)	0.753 (0.548–1.035)	0.858/0.142	1.001 (0.921–1.087)	0.991	0.301
(C>T)	IS	3384/1107/93	*P* = 0.331	*P* = 0.599	*P* = 0.080	0.859/0.141			0.824
rs3743125	Control	2185/2062/416	0.955 (0.890–1.025)	0.940 (0.858–1.029)	0.955 (0.815–1.120)	0.690/0.310	0.984 (0.925–1.048)	0.619	0.024
(G>A)	IS	2178/1998/408	*P* = 0.199	*P* = 0.181	*P* = 0.574	0.693/0.307			0.098

WT, wild type; HT, heterozygote type; MT, mutant type; HWE, Hardy-Weinberg; IS, ischemic stroke. ^a^Adjusted for age, gender, smoking, drinking, hypertension, diabetes, and dyslipidemia. ^b^*P*-value of χ^2^-test for comparison of allele frequencies between the case and control groups.

No significant association of the two tagSNPs at *THBS1* with IS was observed in the case-control study (Supplementary Table 3). The adjusted *OR*s (95%*CI*s) for the addictive model of rs2236741 and rs3743125 were 0.955 (0.872–1.047) and 0.955 (0.890–1.025) after adjustment for covariates. No significant association was observed for the dominant and recessive models of the two SNPs with IS (Table 2). Further stratification analyses by age, gender, smoking, drinking, hypertension, diabetes, and dyslipidemia did not reveal any significant association (Supplementary Tables 4, 5). There was no significant association between *THBS1* variants and TOAST subtypes of IS in the case-control study (Supplementary Table 6).

### Haplotype analyses of rs2236741–rs3743125

Compared with C-G haplotype of rs2236741 and rs3743125, the haplotype C-A, T-G, and T-A were identified to have no significant association with IS (Supplementary Table 7), even after adjustment for covariates [Adjusted *OR*s (95%*CI*s): 0.974 (0.894–1.062), 1.103 (0.829–1.469), and 0.933 (0.845–1.030), respectively] (Table 3). In addition, the haplotypes C-A, T-G, and T-A were not associated with LAA and SVO (Supplementary Table 8).

**TABLE 3:** Haplotype frequencies of rs2236741–rs3743125 and association analyses with ischemic stroke.

Haplotype^a^	All (*n* = 9247)	IS (*n* = 4584)	Control (*n* = 4663)	*OR* (95% *CI*)^c^	*P* ^c^
C-G ^b^	0.678	0.678	0.677	Reference	–
C-A	0.181	0.181	0.182	0.974 (0.894–1.062)	0.668
T-G	0.014	0.015	0.013	1.103 (0.829–1.469)	0.462
T-A	0.127	0.126	0.128	0.933 (0.845–1.030)	0.199

^a^Loci are arranged in the order rs2236741–rs3743125. ^b^C-G was chosen to be the reference. ^c^Adjusted for age, gender, smoking, drinking, hypertension, diabetes, and dyslipidemia.

### Association analyses for the incidence risk of ischemic stroke in the cohort study

After an average of 10.15-year follow-up for 4098 subjects free of stroke at baseline, 319 incident IS cases (10.30%) were observed and the incidence density was 63.08 (per 10^4^ person-year). The variants of rs2236741 and rs3743125 were not associated with the incident risk of IS (Supplementary Table 9), even after adjustment for covariates [Adjusted *HR*s (95%*CI*s) for the addictive model: 1.025 (0.821–1.281) and 0.984 (0.829–1.168)] (Table 4).

**TABLE 4:** Association analyses of *THBS1* variants with the incidence risk of ischemic stroke in the cohort study.

SNP	Genotype	Incident cases	Person-years	Incidence density (/10^4^ person-years)	*HR* (95% *CI*)^a^		
					Additive model	Dominant model	Recessive model
rs2236741	CC	235	37497.42	62.67	1.025 (0.821–1.281)	1.041 (0.811–1.337)	0.915 (0.407–2.056)
	CT	78	11947.78	65.28	*P* = 0.827	*P* = 0.752	*P* = 0.829
	TT	6	1127.20	53.23			
rs3743125	GG	155	23845.56	65.00	0.984 (0.829–1.168)	0.944 (0.758–1.177)	1.097 (0.757–1.589)
	GA	133	21951.53	60.59	*P* = 0.856	*P* = 0.608	*P* = 0.626
	AA	31	4775.31	64.92			

^a^Adjusted for age, gender, smoking, drinking, hypertension, diabetes, and dyslipidemia.

### Association analyses of thrombospondin-1 variants and the risk of long-term death after ischemic stroke

The variants of rs2236741 and rs3743125 were not associated with the long-term death after IS (Supplementary Table 10). Adjusted *HR*s (95%*CI*s) of the addictive model of rs2236741 and rs3743125 for all-cause death, stroke death, IS death, and HS death were 1.014 (0.848–1.212), 1.052 (0.820–1.349), 0.990 (0.723–1.356), 0.948 (0.553–1.626), and adjusted *HR*s (95%*CI*s) of the addictive model of rs3743125 for all-cause death, stroke death, IS death, and HS death were 1.125 (0.982–1.289), 0.985 (0.809–1.199), 1.030 (0.810–1.310), 0.887 (0.583–1.351), respectively. The dominant and recessive models of the two SNPs had no association with the long-term death either (Table 5).

**TABLE 5:** Association analyses of *THBS1* variants with the risk of long-term death after stroke*.

Outcome	SNP	Genotype	Events	Person-years	Density (/10^4^ person-years)	*HR* (95% *CI*)^a^		
						Additive model	Dominant model	Recessive model
All cause death	rs2236741	CC	364	13654.01	266.59	1.014 (0.848–1.212)	0.995 (0.811–1.220)	1.201 (0.691–2.086)
		CT	111	4445.22	249.71	*P* = 0.883	*P* = 0.958	*P* = 0.516
		TT	13	415.70	312.73			
	rs3743125	GG	225	8885.69	253.22	1.125 (0.982–1.289)	1.125 (0.941–1.346)	1.262 (0.943–1.688)
		GA	212	7982.88	265.57	*P* = 0.091	*P* = 0.196	*P* = 0.117
		AA	51	1646.36	309.78			
Stroke death	rs2236741	CC	182	13654.01	133.29	1.052 (0.820–1.349)	1.011 (0.758–1.346)	1.500 (0.740–3.039)
		CT	55	4445.22	123.73	*P* = 0.690	*P* = 0.943	*P* = 0.260
		TT	8	415.70	192.45			
	rs3743125	GG	120	8885.69	135.05	0.985 (0.809–1.199)	0.993 (0.772–1.277)	0.946 (0.598–1.496)
		GA	105	7982.88	131.53	*P* = 0.883	*P* = 0.955	*P* = 0.813
		AA	20	1646.36	121.48			
Ischemic stroke death	rs2236741	CC	122	13654.01	89.35	0.990 (0.723–1.356)	0.940 (0.655–1.349)	1.429 (0.585–3.487)
		CT	34	4445.22	76.49	*P* = 0.952	*P* = 0.738	*P* = 0.433
		TT	5	415.70	120.28			
	rs3743125	GG	77	8885.69	86.66	1.030 (0.810–1.310)	1.047 (0.767–1.429)	1.012 (0.584–1.753)
		GA	70	7982.88	87.69	*P* = 0.808	*P* = 0.773	*P* = 0.967
		AA	14	1646.36	85.04			
Hemorrhagic stroke death	rs2236741	CC	43	13654.01	31.49	0.948 (0.553–1.626)	0.867 (0.466–1.614)	1.630 (0.396–6.708)
		CT	11	4445.22	24.75	*P* = 0.847	*P* = 0.654	*P* = 0.498
		TT	2	415.70	48.11			
	rs3743125	GG	29	8885.69	32.64	0.887 (0.583–1.351)	0.874 (0.517–1.480)	0.820 (0.296–2.272)
		GA	23	7982.88	28.81	*P* = 0.577	*P* = 0.617	*P* = 0.703
		AA	4	1646.36	24.30			

*Ischemic stroke cases aged between 35 and 80 years were selected. ^a^Adjusted for age, gender, smoking, drinking, hypertension, diabetes, and dyslipidemia.

### Comparisons of thrombospondin-1 mRNA expression between ischemic stroke cases and controls

**FIGURE 1:**
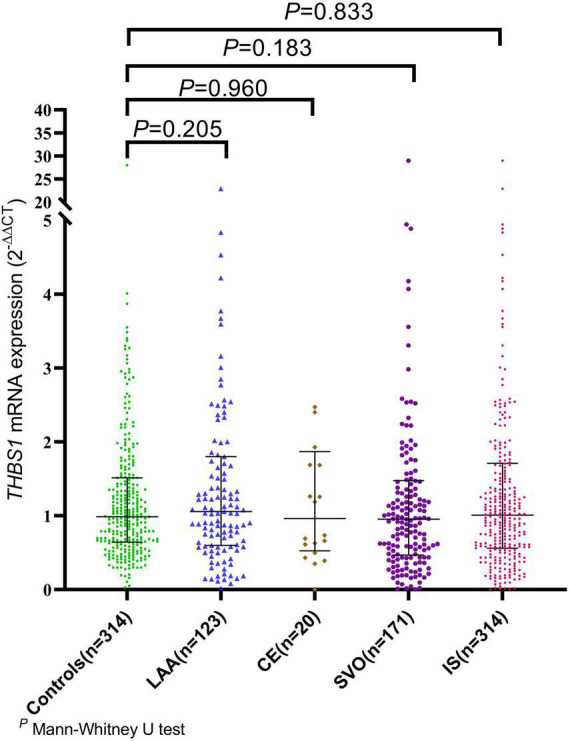
The mRNA expression of *THBS1* common variants in IS cases with different TOAST types and controls. The mRNA expression level of *THBS1* in IS cases was approximately equal to that in controls (1.01 vs. 0.99, *P* = 0.833). Additionally, no significant difference in the *THBS1* mRNA expression was detected between the three TOAST subgroups of IS cases and control group [LAA vs. controls: (1.09 vs. 0.99, *P* = 0.205), CE vs. controls: (0.96 vs. 0.99, *P* = 0.960), SVO vs. controls: (0.96 vs. 0.99, *P* = 0.183)].

**FIGURE 2:**
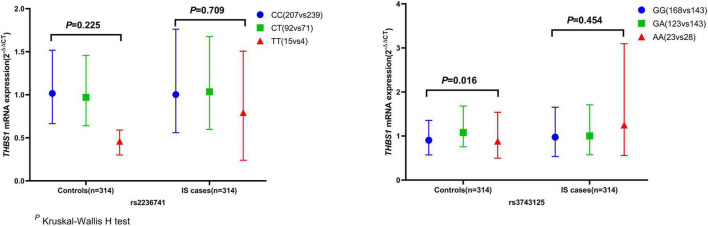
*THBS1* gene mRNA expression (2^– ΔΔCT^) among different genotypes of rs2236741 and rs3743125. The *THBS1* mRNA expression levels significantly differed across rs3743125 GG, GA, and AA carriers in the control group (0.91 vs. 1.08 vs. 0.89, *P* = 0.016) but were not significantly different in IS group (0.97 vs. 1.00 vs. 1.25, *P* = 0.454). Besides, there were no significant differences across rs2236741 CC, CT, and TT carriers in the control group (1.02 vs. 0.97 vs. 0.54, *P* = 0.225) or in the IS cases (1.00 vs. 1.04 vs. 0.79, *P* = 0.709).

The mRNA expression level of *THBS1* in IS cases was approximately equal to that in controls (1.01 vs. 0.99, *P* = 0.833). Further subgroup analyses by age, gender, smoking, drinking, hypertension, diabetes, and dyslipidemia did not observed significant differences in *THBS1* mRNA expression was detected between IS cases and controls (Supplementary Table 11). Additionally, no significant difference in *THBS1* mRNA expression was detected between the three TOAST subgroup of IS and control group (Figure 1). As shown in Figure 2, the *THBS1* mRNA expression levels significantly differed across rs3743125 GG, GA, and AA carriers in the control group (*P* = 0.016), but not in IS group (*P* = 0.454).

### Association analyses of the thrombospondin-1 mRNA expression with the prognosis of ischemic stroke cases

**FIGURE 3:**
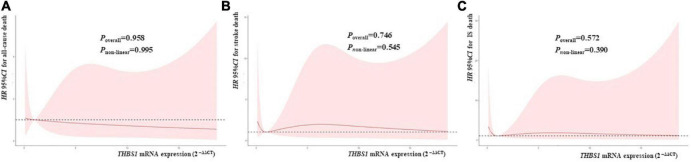
*THBS1* mRNA expression with the *HRs* with 95%CIs for long-term deaths of IS patients. The *HRs* with 95%CIs for all-cause death, stroke death, and IS death had no correlation to *THBS1* mRNA expression in IS patients. (all *P*-values > 0.05).

**FIGURE 4:**
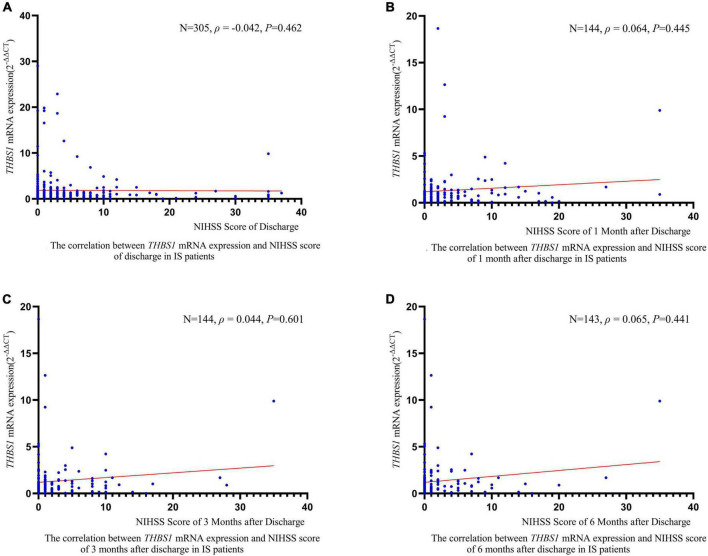
The correlation between *THBS1* mRNA expression and NIHSS scores of different periods after discharge in IS patients. *THBS1* mRNA expression has no significant relevance to NIHSS scores of different periods after discharge in IS patients (all *P*-values > 0.05).

**FIGURE 5:**
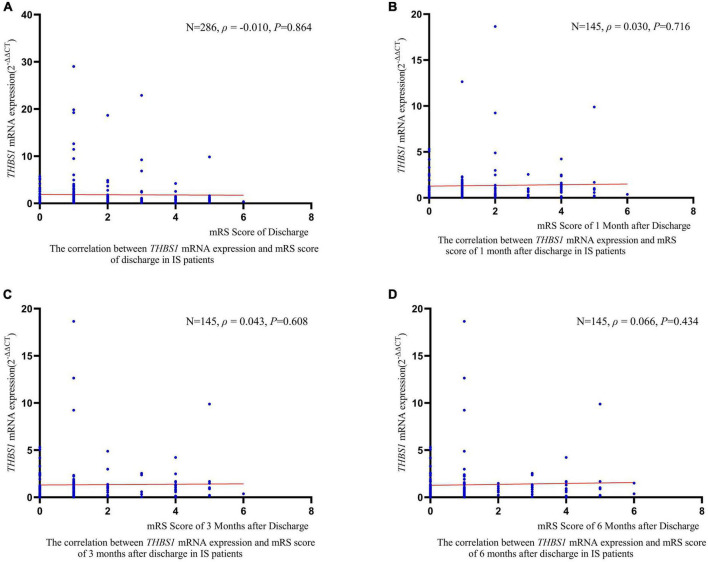
The correlation between *THBS1* mRNA expression and mRS scores of different periods after discharge in IS patients. *THBS1* mRNA expression has no significant relevance to mRS scores of different periods after discharge in IS patients (all *P*-values > 0.05).

RCS regression analyses did not identify significant linear or non-linear correlation between *THBS1* mRNA expression and the risk of all-cause death, stroke death, and IS death in IS patients (Figure 3). The adjusted *HR*s (95%*CI*s) for all-cause death, stroke death, and IS death were 0.962 (0.842–1.099), 0.986 (0.908–1.071), 0.975 (0.821–1.158), respectively (Supplementary Table 12). Furthermore, *THBS1* mRNA expression has no significant relevance to NIHSS scores of discharges (ρ = –0.042, *P* = 0.462), 1 month after discharge (ρ = 0.064, *P* = 0.445), 3 months after discharge (ρ = 0.044, *P* = 0.601), and 6 months after discharge (ρ = 0.065, *P* = 0.441) in IS cases (Figure 4). Similarly, there is no correlation between *THBS1* mRNA expression and mRS scores of discharges (ρ = –0.010, *P* = 0.864), 1 month after discharge (ρ = 0.030, *P* = 0.716), 3 months after discharge (ρ = 0.043, *P* = 0.608), and 6 months after discharge (ρ = 0.066, *P* = 0.434) in IS cases (Figure 5).

## Discussion

In this research, we investigated the association of variations and mRNA expression of *THBS1* with the risk of IS and long-term death after stroke. Our results demonstrated that there was no significant association between tagSNPs of *THBS1* and IS as following aspects. In the case control study, genotype and haplotype analyses identified no significant association with IS and TOAST subtypes of IS. Furthermore, cohort studies did not indicate significant associations between *THBS1* variants and the risk of IS incidence or long-term death after stroke. Previous study showed that the coding polymorphism rs2292305 which is closely linked with rs3743125 we selected (*r*^2^ = 0.932) was identified no significant with cerebral infarction in a Chinese population (Liu et al., 2004). This result was consistent with our results. In addition, the *THBS1* mRNA expression in IS cases was approximately equal to that in controls not only in the total population but also in different subgroups. *THBS1* mRNA expression had no significant association with the prognosis of IS, including NIHSS scores, mRS scores, and long-term deaths after discharge. Thus, there is no significant association of *THBS1* variants and leukocyte mRNA expression with the risk of IS and long-term death after stroke.

Ischemic stroke is one of the most vital causes of neurological morbidity and mortality in the world (Virani et al., 2021). Numerous studies confirmed that the inflammatory reaction that occurs in the cerebral tissue takes part in acute pathologies of ischemic stroke. The function damage to the brain following ischemic stroke results in necrosis and apoptosis; all of these could trigger the inflammatory reaction controlled by the release of ROS, chemokines, and cytokines (Brea et al., 2009). The thrombospondin (THBS) family of secreted matricellular glycoproteins consisting of five members (THBS1-5) are stress and injury mediators of cellular attachment dynamics and extracellular matrix protein production (Schips et al., 2019). Della-Morte et al. (2012) observed that variation in the von Willebrand factor (*VWF*), *THBS1*, and *SERPINE1* genes may involve in the pathogenesis of atherosclerotic plaque and that suggested *THBS1* had the ability to interact with *VWF*, to be an candidate gene for arterial thrombosis (Bonnefoy et al., 2006). Nonetheless, no strong evidence supported the role of *THBS1* in the occurrence of arterial thrombosis (van Schie et al., 2011), and the correlation between peripheral THBS1 levels and long-term outcome of IS (Gao et al., 2015; Al Qawasmeh et al., 2020) merely displayed a concomitant effect rather than sequential relationship of causality. Therefore, the causal relationship between *THBS1* and IS was still lack of sufficient evidence.

Isenberg and Roberts (2020) concluded that the *THBS1* mutation alone may not be sufficient to cause pulmonary artery hypertension (PAH), and *THBS1* was proposed to be a modifier gene for familial PAH. The frequency of common *THBS1* polymorphisms did not differ between PAH and control cohorts (Isenberg and Roberts, 2020). *THBS1* induced lethal cardiac atrophy and played a vital role in intermittent hypoxia-induced fibroblast activation and cardiac fibrosis when overexpressed (Bao et al., 2020; Vanhoutte et al., 2021). Furthermore, hsa-miR-4443 protected atrial fibrillation (AF) by targeting *THBS1* (Xiao et al., 2021), as a biomarker in the development of AF and AF-related complications (Liu et al., 2021). Cardioembolic stroke caused by the embolus forming in the heart and occluding cerebral arteries is rising largely (Maida et al., 2020). AF is one of the risk factors for cardioembolic stroke. A previous study verified that subjects affected by AF have a risk of cerebral ischemia 3–5 times higher than the average (Wolf et al., 1991). Consequently, *THBS1* may have relation to cardioembolic stroke. However, our research did not observe a positive association between *THBS1* and IS not only in the IS case-control study but also in the cohort studies. The reason may be that cardioembolic stroke accounts for only 5.4% of all IS cases in our study.

Although previous study indicated that *THBS1* may related to cardioembolic stroke, there was no significant difference of *THBS1* mRNA expression between CE cases and controls in this study. Consequently, these findings did not support the etiological role of *THBS1* in IS. However, Li et al. found several key genomic expressions (CCL20, *THBS1*, EREG, and IL6 etc.) were dramatically down-regulated in 5 and 24 h after ischemic stroke compared to controls (Li et al., 2017). On the contrary, expression of *THBS1* was noted in the ischemic brain with different temporal expression profiles from different cellular origins after focal cerebral ischemia/reperfusion (Lin et al., 2003). Besides, an animal study suggested that *THBS1* may increase the recruitment of monocytes into the clot and promote the transformation of monocytes into macrophages (Zhou et al., 2010). In advance, it has been reported that *THBS1* could block vascular endothelial growth factor (VEGF)-induced angiogenesis (Iruela-Arispe et al., 1999), so the change of *THBS1* expression in ischemic brains may confer a negative-feedback mechanism in angiogenesis (Kyriakides et al., 1998). In this study, THBS1 mRNA expression levels significantly differed across rs3743125 GG, GA, and AA carriers in the control group but not in IS group. The results may imply the latent biological function of rs3743125 on regulating mRNA expression in the population free of stroke while the effect disappears at IS occurrence. Since no significant association of THBS1 genetic variations and mRNA level was observed with IS or long-term death after IS, the value of this difference is limited to verify the pathogenesis of IS.

Though our current data did not suggest any significant association between the *THBS1* variants or mRNA expression and IS, this research with no doubt has notable strengths. First of all, our study differs from previous *THBS1* relevant studies that focused on the relationship between *THBS1* protein levels in plasma and IS (Gao et al., 2015; Moin et al., 2021). This study is unique since we integrated analyses of the genomic level and the transcriptomic level of *THBS1*. Moreover, we explored the association of *THBS1* candidate SNPs with not only the future new-onset IS but also the long-term outcomes of IS by combining the case-control study and the cohort study. In addition, the large sample size and the experiments with high-quality control ensured the accuracy and reliability of our results. However, some limitations are also worth mentioning. First, we did not detect serum THBS1 protein. Second, we selected candidate SNPs at the *THBS1* gene with the criterion of MAF ≥ 0.05, so could have missed the rare variants with MAF < 0.05 that may have substantial biological effects on the IS occurrence and long-term death after IS.

In conclusion, although these findings need to be validated, our results indicated that there was no significant association between *THBS1* polymorphisms and the risk of IS incidence or long-term death of IS, and no significant difference in *THBS1* mRNA expression observed between IS patients and controls. Our findings, though negative, might as well contribute to verifying that the *THBS1*variants and expression do not affect the susceptibility to IS, and may provide useful evidence and guidance regarding the correlation between THBS1 and IS.

## Data availability statement

The data analyzed in this study is subject to the following licenses/restrictions: “The datasets presented in this article are not available due to participants’ confidentiality.” Requests to access these datasets should be directed to corresponding author.

## Ethics statement

Informed consent was obtained from each participant and this study was approved by the Research Ethics Committee of Nanjing Medical University (#2018571). The patients/participants provided their written informed consent to participate in this study.

## Author contributions

CS designed the study, edited, and proofed the manuscript. CC performed the experiment work, analyzed the data, and wrote the manuscript. XC edited and proofed the manuscript. SY, LW, YJ, LL, and YW collected the data. QL, ZR, JL, and YY collected the samples. XG, FL, and JM performed the experiment work. All authors agreed to be accountable for the content of the work.

## Footnotes

1
https://manticore.niehs.nih.gov/snpinfo/snpfunc.html

2
http://cran.r-project.org/

## References

[B1] Aburima A. Berger M. Spurgeon B. E. J. Webb B. A. Wraith K. S. Febbraio M. (2021). Thrombospondin-1 promotes hemostasis through modulation of cAMP signaling in blood platelets. *Blood* 137 678–689. 10.1182/blood.2020005382 33538796

[B2] Al Qawasmeh M. Alhusban A. Alfwaress F. (2020). An evaluation of the ability of thrombospondin-1 to predict stroke outcomes and mortality after ischemic stroke. *Int. J. Neurosci.* 1–4. [Epub ahead of print]. 10.1080/00207454.2020.1825417 32941082

[B3] August P. Suthanthiran M. (2006). Transforming growth factor beta signaling, vascular remodeling, and hypertension. *N. Engl. J. Med.* 354 2721–2723. 10.1056/NEJMcibr062143 16790709

[B4] Baenziger N. L. Brodie G. N. Majerus P. W. (1972). Isolation and properties of a thrombin-sensitive protein of human platelets. *J. Biol. Chem.* 247 2723–2731. 4260214

[B5] Bao Q. Zhang B. Suo Y. Liu C. Yang Q. Zhang K. (2020). Intermittent hypoxia mediated by TSP1 dependent on STAT3 induces cardiac fibroblast activation and cardiac fibrosis. *Elife* 9:e49923. 10.7554/eLife.49923 31934850 PMC6992386

[B6] Bevan S. Traylor M. Adib-Samii P. Malik R. Paul N. L. Jackson C. (2012). Genetic heritability of ischemic stroke and the contribution of previously reported candidate gene and genomewide associations. *Stroke* 43 3161–3167. 10.1161/strokeaha.112.665760 23042660

[B7] Bonnefoy A. Daenens K. Feys H. B. De Vos R. Vandervoort P. Vermylen J. (2006). Thrombospondin-1 controls vascular platelet recruitment and thrombus adherence in mice by protecting (sub)endothelial VWF from cleavage by ADAMTS13. *Blood* 107 955–964. 10.1182/blood-2004-12-4856 16204318 PMC1895898

[B8] Brea D. Sobrino T. Ramos-Cabrer P. Castillo J. J. C. D. (2009). Inflammatory and neuroimmunomodulatory changes in acute cerebral ischemia. *Cerebrovasc. Dis.* 27 48–64. 10.1159/000200441 19342833

[B9] Campbell B. C. V. De Silva D. A. Macleod M. R. Coutts S. B. Schwamm L. H. Davis S. M. (2019). Ischaemic stroke. *Nat. Rev. Dis. Primers* 5:70. 10.1038/s41572-019-0118-8 31601801

[B10] Contreras-Ruiz L. Ryan D. S. Sia R. K. Bower K. S. Dartt D. A. Masli S. (2014). Polymorphism in THBS1 gene is associated with post-refractive surgery chronic ocular surface inflammation. *Ophthalmology* 121 1389–1397. 10.1016/j.ophtha.2014.01.033 24679443 PMC4197802

[B11] Della-Morte D. Beecham A. Dong C. Wang L. McClendon M. S. Gardener H. (2012). Association between variations in coagulation system genes and carotid plaque. *J. Neurol. Sci.* 323 93–98. 10.1016/j.jns.2012.08.020 22982001 PMC3483411

[B12] Dong J. Yang S. Zhuang Q. Sun J. Wei P. Zhao X. (2021). The Associations of Lipid Profiles With Cardiovascular Diseases and Death in a 10-Year Prospective Cohort Study. *Front. Cardiovasc. Med.* 8:745539. 10.3389/fcvm.2021.745539 34901209 PMC8655628

[B13] Feigin V. L. Nguyen G. Cercy K. Johnson C. O. Alam T. Parmar P. G. (2018). Global, Regional, and Country-Specific Lifetime Risks of Stroke, 1990 and 2016. *N. Engl. J. Med.* 379 2429–2437. 10.1056/NEJMoa1804492 30575491 PMC6247346

[B14] Gao J. B. Tang W. D. Wang H. X. Xu Y. (2015). Predictive value of thrombospondin-1 for outcomes in patients with acute ischemic stroke. *Clin. Chim. Acta* 450 176–180. 10.1016/j.cca.2015.08.014 26296896

[B15] Hong B. B. Chen S. Q. Qi Y. L. Zhu J. W. Lin J. Y. (2015). Association of THBS1 rs1478605 T>C in 5′-untranslated regions with the development and progression of gastric cancer. *Biomed. Rep.* 3 207–214. 10.3892/br.2015.414 26075074 PMC4448071

[B16] Iruela-Arispe M. L. Lombardo M. Krutzsch H. C. Lawler J. Roberts D. D. (1999). Inhibition of angiogenesis by thrombospondin-1 is mediated by 2 independent regions within the type 1 repeats. *Circulation* 100 1423–1431. 10.1161/01.cir.100.13.1423 10500044

[B17] Isenberg J. S. Roberts D. D. (2020). THBS1 (thrombospondin-1). *Atlas Genet. Cytogenet. Oncol. Haematol.* 24 291–299. 10.4267/2042/70774 33244322 PMC7687907

[B18] Isenberg J. S. Romeo M. J. Yu C. Yu C. K. Nghiem K. Monsale J. (2008). Thrombospondin-1 stimulates platelet aggregation by blocking the antithrombotic activity of nitric oxide/cGMP signaling. *Blood* 111 613–623. 10.1182/blood-2007-06-098392 17890448 PMC2200855

[B19] Jacob S. A. Novelli E. M. Isenberg J. S. Garrett M. E. Chu Y. Soldano K. (2017). Thrombospondin-1 gene polymorphism is associated with estimated pulmonary artery pressure in patients with sickle cell anemia. *Am. J. Hematol.* 92 E31–E34. 10.1002/ajh.24635 28033687 PMC5303556

[B20] Jaffe E. A. Ruggiero J. T. Falcone D. J. (1985). Monocytes and macrophages synthesize and secrete thrombospondin. *Blood* 65 79–84. 3965054

[B21] Kyriakides T. R. Zhu Y. H. Smith L. T. Bain S. D. Yang Z. Lin M. T. (1998). Mice that lack thrombospondin 2 display connective tIS casesue abnormalities that are associated with disordered collagen fibrillogenesis, an increased vascular density, and a bleeding diathesis. *J. Cell. Biol.* 140 419–430. 10.1083/jcb.140.2.419 9442117 PMC2132586

[B22] Li W. X. Qi F. Liu J. Q. Li G. H. Dai S. X. Zhang T. (2017). Different impairment of immune and inflammation functions in short and long-term after ischemic stroke. *Am. J. Transl. Res.* 9 736–745. 28337302 PMC5340709

[B23] Lin T. N. Kim G. M. Chen J. J. Cheung W. M. He Y. Y. Hsu C. Y. (2003). Differential regulation of thrombospondin-1 and thrombospondin-2 after focal cerebral ischemia/reperfusion. *Stroke* 34 177–186. 10.1161/01.str.0000047100.84604.ba 12511771

[B24] Liu H. Yang J. Zhang J. Zheng T. Zhai Y. (2021). THBS1: a potential biomarker for atrial fibrillation. *Int. J. Cardiol.* 345:129. 10.1016/j.ijcard.2021.10.152 34757111

[B25] Liu X. N. Song L. Wang D. W. Liao Y. H. Ma A. Q. Zhu Z. M. (2004). [Correlation of thrombospondin-1 G1678A polymorphism to stroke: a study in Chinese population]. *Zhonghua Yi Xue Za Zhi* 84 1959–1962. 15730804

[B26] Maida C. D. Norrito R. L. Daidone M. Tuttolomondo A. Pinto A. (2020). Neuroinflammatory Mechanisms in Ischemic Stroke: Focus on Cardioembolic Stroke, Background, and Therapeutic Approaches. *Int. J. Mol. Sci.* 21:6454. 10.3390/ijms21186454 32899616 PMC7555650

[B27] Matsuo Y. Tanaka M. Yamakage H. Sasaki Y. Muranaka K. Hata H. (2015). Thrombospondin 1 as a novel biological marker of obesity and metabolic syndrome. *Metabolism* 64 1490–1499. 10.1016/j.metabol.2015.07.016 26298466 PMC4936918

[B28] Moin A. S. M. Nandakumar M. Al-Qaissi A. Sathyapalan T. Atkin S. L. Butler A. E. (2021). Potential Biomarkers to Predict Acute Ischemic Stroke in Type 2 Diabetes. *Front. Mol. Biosci.* 8:744459. 10.3389/fmolb.2021.744459 34926573 PMC8671883

[B29] Navarro-Sobrino M. Rosell A. Hernández-Guillamon M. Penalba A. Boada C. Domingues-Montanari S. (2011). A large screening of angiogenesis biomarkers and their association with neurological outcome after ischemic stroke. *Atherosclerosis* 216 205–211. 10.1016/j.atherosclerosis.2011.01.030 21324462

[B30] O’Donnell M. J. Xavier D. Liu L. Zhang H. Chin S. L. Rao-Melacini P. (2010). Risk factors for ischaemic and intracerebral haemorrhagic stroke in 22 countries (the INTERSTROKE study): a case-control study. *Lancet* 376 112–123. 10.1016/s0140-6736(10)60834-3 20561675

[B31] Schaid D. J. Rowland C. M. Tines D. E. Jacobson R. M. Poland G. A. (2002). Score tests for association between traits and haplotypes when linkage phase is ambiguous. *Am. J. Hum. Genet.* 70 425–434. 10.1086/338688 11791212 PMC384917

[B32] Schips T. G. Vanhoutte D. Vo A. Correll R. N. Brody M. J. Khalil H. (2019). Thrombospondin-3 augments injury-induced cardiomyopathy by intracellular integrin inhibition and sarcolemmal instability. *Nat. Commun.* 10:76. 10.1038/s41467-018-08026-8 30622267 PMC6325143

[B33] Sweetwyne M. T. Murphy-Ullrich J. E. (2012). Thrombospondin1 in tissue repair and fibrosis: TGF-β-dependent and independent mechanisms. *Matrix Biol.* 31 178–186. 10.1016/j.matbio.2012.01.006 22266026 PMC3295861

[B34] van Schie M. C. van Loon J. E. de Maat M. P. Leebeek F. W. (2011). Genetic determinants of von Willebrand factor levels and activity in relation to the risk of cardiovascular disease: a review. *J. Thromb. Haemost.* 9 899–908. 10.1111/j.1538-7836.2011.04243.x 21342431

[B35] Vanhoutte D. Schips T. G. Vo A. Grimes K. M. Baldwin T. A. Brody M. J. (2021). Thbs1 induces lethal cardiac atrophy through PERK-ATF4 regulated autophagy. *Nat. Commun.* 12:3928. 10.1038/s41467-021-24215-4 34168130 PMC8225674

[B36] Varma V. Yao-Borengasser A. Bodles A. M. Rasouli N. Phanavanh B. Nolen G. T. (2008). Thrombospondin-1 is an adipokine associated with obesity, adipose inflammation, and insulin resistance. *Diabetes* 57 432–439. 10.2337/db07-0840 18057090 PMC2877915

[B37] Virani S. S. Alonso A. Aparicio H. J. Benjamin E. J. Bittencourt M. S. Callaway C. W. (2021). Heart Disease and Stroke Statistics-2021 Update: A Report From the American Heart Association. *Circulation* 143 e254–e743. 10.1161/cir.0000000000000950 33501848 PMC13036842

[B38] Wolf P. A. Abbott R. D. Kannel W. B. (1991). Atrial fibrillation as an independent risk factor for stroke: the Framingham Study. *Stroke* 22 983–988. 10.1161/01.str.22.8.983 1866765

[B39] Xiao J. Zhang Y. Tang Y. Dai H. OuYang Y. Li C. (2021). hsa-miR-4443 inhibits myocardial fibroblast proliferation by targeting THBS1 to regulate TGF-β1/α-SMA/collagen signaling in atrial fibrillation. *Braz. J. Med. Biol. Res.* 54:e10692. 10.1590/1414-431x202010692 33681892 PMC7931814

[B40] Zhou H. J. Zhang H. N. Tang T. Zhong J. H. Qi Y. Luo J. K. (2010). Alteration of thrombospondin-1 and –2 in rat brains following experimental intracerebral hemorrhage. Laboratory investigation. *J. Neurosurg.* 113 820–825. 10.3171/2010.1.Jns09637 20136391

