# Supplementary material for: Association of THBS1 genetic variants and mRNA expression with the risks of ischemic stroke and long-term death after stroke
Source: Front Aging Neurosci. 2022 Sep 23;14:1006473. doi: 10.3389/fnagi.2022.1006473 (PMC9545898; doi:10.3389/fnagi.2022.1006473)
Supplement: Supplementary file 1 [file Data_Sheet_1.pdf]

Supplementary Material

Table S1 Biological information and function prediction for selected tagSNPs in *THBS1*

SNP	Chromosome	Allele	Position	Enhancer	TFBS	eQTL	Nearby Gene	MAF
rs2236741	15	C/T	Intron variant	Y	Y	Y	THBS1	0.833
rs3743125	15	G/A	3'-UTR variant	Y	-	Y	THBS1	0.685

SNP, single nucleotide polymorphism; TFBS, transcription factor binding site; eQTL, expression quantitative trait loci; MAF, minor allele frequency.

Table S2 Demographic and clinical characteristics of the study population in the cohort study

Characteristics	Group	Follow-up study for IS				Follow-up study for the prognosis of IS				
		Non-IS (n=3779)	New IS (n=319)	Z/χ^2	*P*	IS cases* (n=3158)	All-cause death (n=488)	Stroke death (n=245)	IS death (n=161)	HS death (n=56)
Age (year)		58 (52, 66)	68 (61, 75)	12.957	<0.001[a]	66 (59, 71)	71 (66, 76)	71 (66, 76)	71 (67, 76)	70 (64, 73)
Gender [n (%)]	Male	1522 (40.3)	141 (44.2)	1.880	0.170[b]	1921 (60.8)	307 (62.9)	157 (64.1)	103 (64.0)	36 (64.3)
	Female	2257 (59.7)	178 (55.8)			1237 (39.2)	181 (37.1)	88 (35.9)	58 (36.0)	20 (35.7)
SBP (mmHg)		133 (123, 140)	138 (127, 146)	4.697	<0.001[a]	150 (136, 164)	150 (138, 167)	150 (140, 170)	150 (140, 170)	150 (140, 170)
DBP (mmHg)		82 (78, 89)	83 (79, 90)	1.722	0.085[a]	88 (80, 96)	90 (80, 96)	90 (80, 100)	90 (80, 100)	90 (80, 99)
GLU (mmol/L)		5.27 (4.85, 5.79)	5.33 (4.85, 6.00)	1.308	0.191[a]	4.75 (4.31, 5.32)	4.66 (4.28, 5.28)	4.64 (4.29, 5.30)	4.62 (4.29, 5.28)	4.79 (4.30, 5.41)
TC (mmol/L)		4.78 (4.21, 5.44)	5.02 (4.39, 5.65)	3.627	<0.001[a]	4.60 (3.94, 5.34)	4.52 (3.79, 5.22)	4.57 (3.78, 5.15)	4.59 (3.79, 5.13)	4.19 (3.73, 5.25)
TG (mmol/L)		1.32 (0.89, 2.00)	1.43 (1.01, 2.07)	2.508	0.012[a]	1.44 (1.01, 2.12)	1.24 (0.87, 1.90)	1.27 (0.86, 1.91)	1.22 (0.86, 1.85)	1.45 (0.84, 2.00)
HDL-C (mmol/L)		1.33 (1.13, 1.55)	1.35 (1.18, 1.54)	0.622	0.534[a]	1.13 (0.97, 1.33)	1.12 (0.95, 1.33)	1.13 (0.98, 1.32)	1.13 (0.99, 1.33)	1.14 (0.97, 1.34)
LDL-C (mmol/L)		2.64 (2.19, 3.10)	2.79 (2.32, 3.32)	3.715	<0.001[a]	2.62 (2.11, 3.15)	2.55 (2.05, 3.15)	2.61 (2.09, 3.14)	2.73 (2.16, 3.14)	2.37 (1.93, 2.90)
Smoking [n (%)]	No	2863 (75.8)	240 (75.2)	0.044	0.833[b]	2307 (73.1)	377 (77.3)	185 (75.5)	126 (78.3)	40 (71.4)
	Yes	916 (24.2)	79 (24.8)			851 (26.9)	111 (22.7)	60 (24.5)	35 (21.7)	16 (28.6)
Drinking [n (%)]	No	2958 (78.3)	257 (80.6)	0.912	0.340[b]	2635 (83.4)	430 (88.1)	215 (87.8)	142 (88.2)	50 (89.3)
	Yes	821 (21.7)	62 (19.4)			523 (16.6)	58 (11.9)	30 (12.2)	19 (11.8)	6 (10.7)
Hypertension	No	2002 (53.0)	108 (33.9)	43.060	<0.001[b]	517 (16.4)	65 (13.3)	30 (12.2)	19 (11.8)	6 (10.7)
[n (%)]	Yes	1777 (47.0)	211 (66.1)			2641 (83.6)	423 (86.7)	215 (87.8)	142 (88.2)	50 (89.3)
Diabetes [n (%)]	No	3369 (89.2)	265 (83.1)	10.825	0.001[b]	2298 (72.8)	346 (70.9)	185 (75.5)	125 (77.6)	40 (71.4)
	Yes	410 (10.8)	54 (16.9)			860 (27.2)	142 (29.1)	60 (24.5)	36 (22.4)	16 (28.6)
Dyslipidemia	No	1537 (40.7)	105 (32.9)	7.371	0.007[b]	1863 (59.0)	254 (52.0)	139 (56.7)	95 (59.0)	28 (50.0)
[n (%)]	Yes	2242 (59.3)	214 (67.1)			1295 (41.0)	234 (48.0)	106 (43.3)	66 (41.0)	28 (50.0)

*[a] Mann-Whitney U test; [b]χ^2 test; IS, ischemic stroke; SBP, systolic blood pressure; DBP, diastolic blood pressure; GLU, glucose; TC, total cholesterol; TG, triglyceride; HDL-C, high-density lipoprotein-cholesterol; LDL-C, low-density lipoprotein-cholesterol. * Ischemic stroke cases aged between 35 and 80 years were selected.*

Table S3 Association analyses of *THBS1* variants and the risk of ischemic stroke in the case-control study

SNP	Group	WT/HT/MT	*OR* (95% *CI*)			Allele			*P* for
			Additive model	Dominant model	Recessive model	Major/Minor	*OR* (95% *CI*)	*P* [a]	*HWE*
rs2236741	Control	3445/1116/102	0.996 (0.917-1.081)	1.003 (0.914-1.100)	0.926 (0.697-1.230)	0.858/0.142	1.001 (0.921-1.087)	0.991	0.301
(C>T)	IS	3384/1107/93	*P*=0.922	*P*=0.950	*P*=0.596	0.859/0.141			0.824
rs3743125	Control	2185/2062/416	0.984 (0.923-1.048)	0.974 (0.898-1.057)	0.997 (0.864-1.151)	0.690/0.310	0.984 (0.925-1.048)	0.619	0.024
(G>A)	IS	2178/1998/408	*P*=0.614	*P*=0.528	*P*=0.972	0.693/0.307			0.098

WT, wild type; HT, heterozygote type; MT, mutant type; HWE, Hardy-Weinberg; IS, ischemic stroke.

[a] *P value of* χ^2 *test for comparison of allele frequencies between the case and control groups.*

Table S4 Stratification analyses for the association of *THBS1* variants and the risk of ischemic stroke

SNP	Factor	Stratum	Group	WT/HT/MT	*OR* (95% *CI*)		
					Additive model	Dominant model	Recessive model
rs2236741(C>T)	Age	≤65 years	Control	1554/546/46	1.009 (0.894-1.138)	1.010 (0.882-1.157)	1.012 (0.668-1.533)
			IS	1541/518/45	*P*=0.885	*P*=0.885	*P*=0.955
		>65 years	Control	1891/570/56	0.985 (0.880-1.103)	0.997 (0.878-1.133)	0.857 (0.581-1.265)
			IS	1843/589/48	*P*=0.794	*P*=0.969	*P*=0.438
	Gender	Male	Control	1398/495/50	0.934 (0.833-1.047)	0.927 (0.814-1.057)	0.895 (0.615-1.304)
			IS	2001/658/63	*P*=0.243	*P*=0.259	*P*=0.564
		Female	Control	2047/621/52	1.027 (0.909-1.161)	1.050 (0.917-1.203)	0.841 (0.534-1.323)
			IS	1383/449/30	*P*=0.665	*P*=0.477	*P*=0.453
	Smoking	No	Control	2761/884/80	1.002 (0.913-1.099)	1.009 (0.909-1.120)	0.938 (0.680-1.294)
			IS	2640/858/72	*P*=0.972	*P*=0.868	*P*=0.696
		Yes	Control	684/232/22	0.971 (0.813-1.160)	0.977 (0.800-1.194)	0.880 (0.481-1.612)
			IS	744/249/21	*P*=0.749	*P*=0.822	*P*=0.680
	Drinking	No	Control	2801/873/74	1.042 (0.951-1.141)	1.053 (0.951-1.167)	0.996 (0.722-1.373)
			IS	2926/964/78	*P*=0.378	*P*=0.319	*P*=0.978
		Yes	Control	644/243/28	0.841 (0.689-1.027)	0.820 (0.651-1.032)	0.791 (0.419-1.493)
			IS	458/143/15	*P*=0.090	*P*=0.090	*P*=0.469
	Hypertension	No	Control	1743/545/39	1.076 (0.907-1.276)	1.103 (0.914-1.331)	0.893 (0.455-1.752)
			IS	536/187/11	*P*=0.403	*P*=0.309	*P*=0.741
		Yes	Control	1702/571/63	0.936 (0.845-1.036)	0.944 (0.841-1.061)	0.785 (0.563-1.095)
			IS	2848/920/82	*P*=0.202	*P*=0.334	*P*=0.154
	Diabetes	No	Control	2971/947/82	1.002 (0.913-1.101)	1.014 (0.913-1.127)	0.893 (0.639-1.248)
			IS	2462/804/61	*P*=0.960	*P*=0.789	*P*=0.507
		Yes	Control	474/169/20	0.916 (0.764-1.099)	0.911 (0.739-1.124)	0.840 (0.476-1.480)
			IS	922/303/32	*P*=0.346	*P*=0.385	*P*=0.546
	Dyslipidemia	No	Control	2141/684/66	1.024 (0.922-1.138)	1.046 (0.928-1.178)	0.876 (0.609-1.260)
			IS	1971/668/54	*P*=0.657	*P*=0.462	*P*=0.476
		Yes	Control	1304/432/36	0.955 (0.838-1.090)	0.943 (0.813-1.093)	1.015 (0.643-1.605)
			IS	1413/439/39	*P*=0.498	*P*=0.433	*P*=0.948

	Age	≤65 years	Control	986/952/208	1.002 (0.913-1.099)	1.002 (0.889-1.130)	1.003 (0.815-1.235)
rs3743125(G>A)			IS	993/919/192	*P*=0.971	*P*=0.976	*P*=0.976
		>65 years	Control	1199/1110/208	0.969 (0.888-1.056)	0.951 (0.850-1.063)	0.993 (0.815-1.209)
			IS	1185/1079/216	*P*=0.470	*P*=0.374	*P*=0.944
	Gender	Male	Control	903/862/178	0.968 (0.885-1.059)	0.958 (0.852-1.076)	0.966 (0.789-1.184)
			IS	1297/1183/242	*P*=0.479	*P*=0.469	*P*=0.741
		Female	Control	1282/1200/238	0.998 (0.911-1.094)	0.990 (0.880-1.114)	1.022 (0.830-1.257)
			IS	881/815/166	*P*=0.966	*P*=0.866	*P*=0.840
	Smoking	No	Control	1749/1635/341	0.995 (0.927-1.068)	0.992 (0.905-1.088)	0.997 (0.850-1.169)
			IS	1683/1561/326	*P*=0.888	*P*=0.871	*P*=0.973
		Yes	Control	436/427/75	0.945 (0.822-1.088)	0.911 (0.762-1.088)	1.012 (0.730-1.403)
			IS	495/437/82	*P*=0.434	*P*=0.302	*P*=0.941
	Drinking	No	Control	1764/1655/329	1.007 (0.940-1.080)	1.006 (0.920-1.100)	1.021 (0.873-1.195)
			IS	1862/1751/355	*P*=0.833	*P*=0.902	*P*=0.795
		Yes	Control	421/407/87	0.862 (0.736-1.011)	0.809 (0.659-0.993)	0.896 (0.626-1.281)
			IS	316/247/53	*P*=0.067	*P*=0.042	*P*=0.547
	Hypertension	No	Control	1116/1017/194	0.993 (0.872-1.130)	0.968 (0.820-1.143)	1.068 (0.796-1.433)
			IS	358/311/65	*P*=0.913	*P*=0.700	*P*=0.660
		Yes	Control	1069/1045/222	0.951 (0.878-1.029)	0.941 (0.849-1.043)	0.931 (0.780-1.111)
			IS	1820/1687/343	*P*=0.213	*P*=0.249	*P*=0.428
	Diabetes	No	Control	1877/1773/350	0.978 (0.911-1.051)	0.961 (0.877-1.054)	1.011 (0.859-1.189)
			IS	1594/1439/294	*P*=0.551	*P*=0.400	*P*=0.896
		Yes	Control	308/289/66	0.979 (0.847-1.132)	1.000 (0.828-1.208)	0.902 (0.656-1.241)
			IS	584/559/114	*P*=0.775	*P*=1.000	*P*=0.527
	Dyslipidemia	No	Control	1360/1272/259	1.029 (0.949-1.116)	1.027 (0.924-1.141)	1.068 (0.891-1.280)
			IS	1249/1188/256	*P*=0.485	*P*=0.620	*P*=0.480
		Yes	Control	825/790/157	0.920 (0.831-1.018)	0.902 (0.792-1.027)	0.899 (0.712-1.135)
			IS	929/810/152	*P*=0.108	*P*=0.120	*P*=0.371

WT, wild type; HT, heterozygote; MT, mutant type.

Table S5 Stratification analyses for the association of *THBS1* variants and the risk of ischemic stroke after adjustment for covariates

SNP	Factor	Stratum	Group	WT/HT/MT	*OR* (95% *CI*)		
					Additive model	Dominant model	Recessive model
rs2236741(C>T)	Age	≤65 years	Control	1554/546/46	1.001 (0.872-1.148)	1.024 (0.878-1.194)	0.797 (0.492-1.291)
			IS	1541/518/45	*P*=0.992[a]	*P*=0.761[a]	*P*=0.357[a]
		>65 years	Control	1891/570/56	0.922 (0.815-1.044)	0.937 (0.815-1.077)	0.705 (0.462-1.078)
			IS	1843/589/48	*P*=0.201[a]	*P*=0.361[a]	*P*=0.107[a]
	Gender	Male	Control	1398/495/50	0.910 (0.800-1.035)	0.915 (0.790-1.059)	0.759 (0.498-1.156)
			IS	2001/658/63	*P*=0.151[b]	*P*=0.233[b]	*P*=0.199[b]
		Female	Control	2047/621/52	1.014 (0.888-1.158)	1.043 (0.900-1.209)	0.757 (0.464-1.237)
			IS	1383/449/30	*P*=0.840[b]	*P*=0.572[b]	*P*=0.267[b]
	Smoking	No	Control	2761/884/80	0.955 (0.860-1.060)	0.972 (0.864-1.093)	0.753 (0.525-1.080)
			IS	2640/858/72	*P*=0.384[c]	*P*=0.632[c]	*P*=0.123[c]
		Yes	Control	684/232/22	0.982 (0.803-1.201)	1.010 (0.805-1.268)	0.736 (0.372-1.457)
			IS	744/249/21	*P*=0.857[c]	*P*=0.931[c]	*P*=0.380[c]
	Drinking	No	Control	2801/873/74	0.978 (0.883-1.083)	0.997 (0.890-1.118)	0.774 (0.539-1.112)
			IS	2926/964/78	*P*=0.664[d]	*P*=0.965[d]	*P*=0.159[d]
		Yes	Control	644/243/28	0.876 (0.703-1.092)	0.874 (0.678-1.128)	0.730 (0.364-1.461)
			IS	458/143/15	*P*=0.240[d]	*P*=0.302[d]	*P*=0.374[d]
	Hypertension	No	Control	1743/545/39	1.098 (0.917-1.316)	1.110 (0.911-1.353)	1.106 (0.547-2.233)
			IS	536/187/11	*P*=0.309[e]	*P*=0.301[e]	*P*=0.780[e]
		Yes	Control	1702/571/63	0.913 (0.822-1.015)	0.927 (0.822-1.045)	0.706 (0.499-0.998)
			IS	2848/920/82	*P*=0.092[e]	*P*=0.215[e]	*P*=0.049[e]
	Diabetes	No	Control	2971/947/82	0.986 (0.888-1.094)	1.000 (0.891-1.124)	0.829 (0.572-1.201)
			IS	2462/804/61	*P*=0.784[f]	*P*=0.995[f]	*P*=0.321[f]
		Yes	Control	474/169/20	0.867 (0.714-1.053)	0.884 (0.706-1.107)	0.614 (0.338-1.115)
			IS	922/303/32	*P*=0.151[f]	*P*=0.282[f]	*P*=0.109[f]
	Dyslipidemia	No	Control	2141/684/66	0.976 (0.867-1.098)	1.003 (0.879-1.146)	0.725 (0.484-1.087)
			IS	1971/668/54	*P*=0.682[g]	*P*=0.959[g]	*P*=0.119[g]
		Yes	Control	1304/432/36	0.930 (0.802-1.077)	0.932 (0.790-1.099)	0.815 (0.485-1.368)

			IS	1413/439/39	*P*=0.332[g]	*P*=0.401[g]	*P*=0.439[g]
rs3743125(G>A)	Age	≤65 years	Control	986/952/208	0.972 (0.876-1.079)	0.975 (0.851-1.116)	0.976 (0.772-1.233)
			IS	993/919/192	*P*=0.597[a]	*P*=0.570[a]	*P*=0.838[a]
		>65 years	Control	1199/1110/208	0.935 (0.850-1.028)	0.905 (0.801-1.023)	0.931 (0.750-1.155)
			IS	1185/1079/216	*P*=0.166[a]	*P*=0.158[a]	*P*=0.516[a]
	Gender	Male	Control	903/862/178	0.932 (0.843-1.031)	0.911 (0.799-1.038)	0.928 (0.740-1.164)
			IS	1297/1183/242	*P*=0.174[b]	*P*=0.166[b]	*P*=0.516[b]
		Female	Control	1282/1200/238	0.976 (0.883-1.079)	0.966 (0.850-1.099)	0.982 (0.783-1.231)
			IS	881/815/166	*P*=0.634[b]	*P*=0.602[b]	*P*=0.874[b]
	Smoking	No	Control	1749/1635/341	0.967 (0.893-1.046)	0.962 (0.868-1.066)	0.947 (0.793-1.131)
			IS	1683/1561/326	*P*=0.400[c]	*P*=0.457[c]	*P*=0.549[c]
		Yes	Control	436/427/75	0.936 (0.798-1.097)	0.881 (0.720-1.078)	1.054 (0.730-1.521)
			IS	495/437/82	*P*=0.411[c]	*P*=0.230[c]	*P*=0.779[c]
	Drinking	No	Control	1764/1655/329	0.982 (0.909-1.061)	0.979 (0.886-1.081)	0.974 (0.817-1.160)
			IS	1862/1751/355	*P*=0.646[d]	*P*=0.674[d]	*P*=0.765[d]
		Yes	Control	421/407/87	0.868 (0.728-1.035)	0.801 (0.638-1.005)	0.952 (0.642-1.412)
			IS	316/247/53	*P*=0.115[d]	*P*=0.056[d]	*P*=0.807[d]
	Hypertension	No	Control	1116/1017/194	0.987 (0.861-1.131)	0.968 (0.813-1.153)	1.036 (0.759-1.414)
			IS	358/311/65	*P*=0.849[e]	*P*=0.713[e]	*P*=0.824[e]
		Yes	Control	1069/1045/222	0.942 (0.868-1.023)	0.929 (0.835-1.034)	0.924 (0.769-1.110)
			IS	1820/1687/343	*P*=0.156[e]	*P*=0.178[e]	*P*=0.399[e]
	Diabetes	No	Control	1877/1773/350	0.956 (0.883-1.035)	0.931 (0.841-1.031)	0.990 (0.827-1.184)
			IS	1594/1439/294	*P*=0.268[f]	*P*=0.173[f]	*P*=0.910[f]
		Yes	Control	308/289/66	0.970 (0.831-1.132)	0.993 (0.812-1.215)	0.879 (0.626-1.233)
			IS	584/559/114	*P*=0.698[f]	*P*=0.974[f]	*P*=0.454[f]
	Dyslipidemia	No	Control	1360/1272/259	0.993 (0.907-1.088)	0.968 (0.860-1.088)	1.065 (0.871-1.304)
			IS	1249/1188/256	*P*=0.886[g]	*P*=0.587[g]	*P*=0.539[g]
		Yes	Control	825/790/157	0.910 (0.813-1.018)	0.911 (0.789-1.052)	0.819 (0.633-1.060)
			IS	929/810/152	*P*=0.098[g]	*P*=0.205[g]	*P*=0.129[g]

WT, wild type; HT, heterozygote; MT, mutant type.

[a] *Adjusted for gender, smoking, drinking, hypertension, diabetes, and dyslipidemia.*

[b] *Adjusted for age, smoking, drinking, hypertension, diabetes, and dyslipidemia.*

[c] *Adjusted for age, gender, drinking, hypertension, diabetes, and dyslipidemia.*

[d] *Adjusted for age, gender, smoking, hypertension, diabetes, and dyslipidemia.*

[e] *Adjusted for age, gender, smoking, drinking, diabetes, and dyslipidemia.*

[f] *Adjusted for age, gender, smoking, drinking, hypertension, and dyslipidemia.*

[g] *Adjusted for age, gender, smoking, drinking, hypertension, and diabetes.*

Table S6 Association analyses of *THBS1* variants and the risk of TOAST subtypes of IS in the case-control study

SNP	Group	WT/HT/MT	*OR* (95% *CI*)			*OR* (95% *CI*) [a]		
			Additive model	Dominant model	Recessive model	Additive model	Dominant model	Recessive model
rs2236741	Control	3445/1116/102	1.033(0.923-1.157)	1.044(0.919-1.186)	0.987(0.669-1.455)	0.962(0.850-1.089)	0.983(0.854-1.130)	0.752(0.491-1.154)
(C>T)	LAA	1184/402/35	*P*=0.570	*P*=0.507	*P*=0.947	*P*=0.543	*P*=0.806	*P*=0.192
	Control	3445/1116/102	0.987(0.887-1.098)	0.999(0.886-1.126)	0.859(0.588-1.257)	0.935(0.832-1.051)	0.951(0.833-1.084)	0.726(0.481-1.095)
	SVO	1450/475/37	*P*=0.807	*P*=0.983	*P*=0.435	*P*=0.262	*P*=0.449	*P*=0.126
rs3743125	Control	2185/2062/416	1.044(0.956-1.139)	1.042(0.930-1.167)	1.095(0.903-1.328)	1.007(0.915-1.108)	0.996(0.880-1.128)	1.045(0.846-1.291)
(G>A)	LAA	743/721/157	*P*=0.337	*P*=0.478	*P*=0.357	*P*=0.890	*P*=0.952	*P*=0.680
	Control	2185/2062/416	0.966(0.890-1.049)	0.957(0.861-1.063)	0.963(0.798-1.161)	0.933(0.853-1.021)	0.916(0.815-1.028)	0.921(0.752-1.129)
	SVO	941/852/169	*P*=0.416	*P*=0.411	*P*=0.689	*P*=0.131	*P*=0.135	*P*=0.430

WT, wild type; HT, heterozygote type; MT, mutant type; HWE, Hardy-Weinberg; IS, ischemic stroke; LAA: large-artery atherosclerosis; SVO: small-vessel occlusion. [a] *Adjusted for age, gender, smoking, drinking, hypertension, diabetes and dyslipidemia.*

Table S7 Haplotype frequencies of rs2236741-rs3743125 and association analyses with ischemic stroke

Haplotype [a]	All (n=9247)	IS (n=4584)	Control (n=4663)	*OR* (95% *CI*)	*P*
C-G [b]	0.678	0.678	0.677	Reference	-
C-A	0.181	0.181	0.182	0.992 (0.918-1.073)	0.821
T-G	0.014	0.015	0.013	1.131 (0.875-1.463)	0.333
T-A	0.127	0.126	0.128	0.979 (0.896-1.070)	0.662

[a] Loci are arranged in the order rs2236741-rs3743125. [b] C-G was chosen to be the reference.

Table S8 Haplotype frequencies of rs2236741-rs3743125 and association analyses with TOAST subtypes of IS

TOAST subtype	Haplotype [a]	All(n=6284)	Control(n=4663)	Cases	*OR* (95% *CI*)	*P*	*OR* (95% *CI*) [c]	P^c
LAA(n=1621)	C-G [b]	0.674	0.677	0.663	Reference	-	Reference	-
	C-A	0.184	0.182	0.192	1.078(0.969-1.199)	0.250	1.063(0.946-1.063)	0.287
	T-G	0.014	0.013	0.018	1.360(0.984-1.880)	0.087	1.244(0.870-1.779)	0.286
	T-A	0.128	0.128	0.127	1.015(0.897-1.148)	0.999	0.944(0.825-1.080)	0.303
SVO(n=1962)	C-G [b]	0.678	0.677	0.680	Reference	-	Reference	-
	C-A	0.181	0.182	0.180	0.981(0.851-1.073)	0.710	0.959(0.858-1.071)	0.581
	T-G	0.014	0.013	0.016	1.214(0.887-1.662)	0.207	1.119(0.791-1.585)	0.474
	T-A	0.127	0.128	0.124	0.956(0.851-1.073)	0.485	0.904(0.797-1.026)	0.171

[a] *Loci are arranged in the order rs2236741-rs3743125; IS, ischemic stroke; LAA: large-artery atherosclerosis; SVO: small-vessel occlusion.*

[b] *C-G was chosen to be the reference.*

[c] *Adjusted for age, gender, smoking, drinking, hypertension, diabetes and dyslipidemia.*

Table S9 Association analyses of *THBS1* variants with the incidence risk of IS in the cohort study

SNP	Genotype	Incident cases	Person-years	Incidence density (/10^4 Person-years)	*HR* (95% *CI*)		
					Additive model	Dominant model	Recessive model
rs2236741	CC	235	37497.42	62.67	1.006 (0.807-1.255)	1.027 (0.800-1.317)	0.842 (0.375-1.889)
	CT	78	11947.78	65.28	*P*=0.955	*P*=0.837	*P*=0.677
	TT	6	1127.20	53.23			
rs3743125	GG	155	23845.56	65.00	0.974 (0.822-1.154)	0.945 (0.759-1.177)	1.034 (0.714-1.498)
	GA	133	21951.53	60.59	*P*=0.759	*P*=0.613	*P*=0.858
	AA	31	4775.31	64.92			

Table S10 Association analyses of *THBS1* variants with the risk of long-term death after stroke*

Outcome	SNP	Genotype	Events	Person-years	Density (/10^4 Person-years)	*HR* (95% *CI*) Additive model	Dominant model	Recessive model
All-cause death	rs2236741	CC	364	13654.01	266.59	0.982 (0.821-1.175)	0.957 (0.780-1.173)	1.190 (0.686-2.064)
		CT	111	4445.22	249.71	*P*=0.845	*P*=0.670	*P*=0.537
		TT	13	415.70	312.73			
	rs3743125	GG	225	8885.69	253.22	1.088 (0.950-1.246)	1.081 (0.905-1.292)	1.204 (0.901-1.609)
		GA	212	7982.88	265.57	*P*=0.222	*P*=0.390	*P*=0.210
		AA	51	1646.36	309.78			
Stroke death	rs2236741	CC	182	13654.01	133.29	1.021 (0.796-1.310)	0.974 (0.731-1.297)	1.470 (0.727-2.975)
		CT	55	4445.22	123.73	*P*=0.871	*P*=0.858	*P*=0.284
		TT	8	415.70	192.45			
	rs3743125	GG	120	8885.69	135.05	0.960 (0.789-1.167)	0.961 (0.748-1.235)	0.912 (0.577-1.441)
		GA	105	7982.88	131.53	*P*=0.682	*P*=0.758	*P*=0.693
		AA	20	1646.36	121.48			
Ischemic stroke death	rs2236741	CC	122	13654.01	89.35	0.955 (0.696-1.309)	0.899 (0.627-1.290)	1.401 (0.575-3.414)
		CT	34	4445.22	76.49	*P*=0.773	*P*=0.564	*P*=0.458
		TT	5	415.70	120.28			
	rs3743125	GG	77	8885.69	86.66	0.999 (0.786-1.270)	1.006 (0.738-1.370)	0.977 (0.565-1.691)
		GA	70	7982.88	87.69	*P*=0.994	*P*=0.971	*P*=0.935
		AA	14	1646.36	85.04			
Hemorrhagic stroke death	rs2236741	CC	43	13654.01	31.49	0.932 (0.544-1.599)	0.850 (0.457-1.580)	1.617 (0.394-6.633)
		CT	11	4445.22	24.75	*P*=0.799	*P*=0.607	*P*=0.504
		TT	2	415.70	48.11			
	rs3743125	GG	29	8885.69	32.64	0.872 (0.575-1.325)	0.859 (0.509-1.452)	0.789 (0.285-2.181)
		GA	23	7982.88	28.81	*P*=0.522	*P*=0.571	*P*=0.648
		AA	4	1646.36	24.30			

* *Ischemic stroke cases aged between 35 and 80 years were selected.*

Table S11 Comparisons of *THBS1* mRNA expression between IS cases and controls in subgroups

Factor	Stratum	Control(n)	IS(n)	P^a
mRNA expression($2^{-\Delta\Delta CT}$)		0.99 (0.64, 1.51) (n=314)	1.01 (0.56, 1.71) (n=314)	0.833
Age	≤65 years	1.01 (0.67, 1.44) (148)	0.57 (0.60, 1.65) (135)	0.525
	>65 years	0.98 (0.60, 1.52) (166)	1.03 (0.56, 1.74) (179)	0.815
	P^a	0.620	0.579	
Gender	Male	0.98 (0.64, 1.56) (182)	0.97 (0.49, 1.62) (182)	0.469
	Female	1.01 (0.65, 1.43) (132)	1.08 (0.59, 1.81) (132)	0.597
	P^a	0.929	0.304	
Smoking	No	0.98 (0.63, 1.44) (232)	0.96 (0.53, 1.69) (266)	0.835
	Yes	1.05 (0.67, 1.65) (82)	1.11 (0.60, 1.81) (48)	0.707
	P^a	0.297	0.302	
Drinking	No	0.98 (0.62, 1.43) (220)	0.99 (0.53, 1.72) (283)	0.965
	Yes	1.04 (0.66, 1.75) (94)	1.04 (0.73, 1.58) (31)	0.873
	P^a	0.248	0.548	
Hypertension	No	1.06 (0.64, 1.53) (85)	0.96 (0.47, 1.32) (42)	0.298
	Yes	0.97 (0.64, 1.51) (229)	1.03 (0.57, 1.79) (272)	0.893
	P^a	0.700	0.481	
Diabetes	No	1.00 (0.65, 1.53) (236)	1.03 (0.58, 1.80) (208)	0.991
	Yes	0.94 (0.61, 1.43) (78)	0.92 (0.53, 1.52) (106)	0.911
	P^a	0.464	0.438	
Dyslipidemia	No	1.02 (0.65, 1.54) (152)	1.02 (0.54, 1.73) (241)	0.825
	Yes	0.98 (0.61, 1.51) (162)	0.94 (0.58, 1.56) (73)	0.792
	P^a	0.662	0.711	

[a] *Mann-Whitney U test*

Table S12 Association analyses of *THBS1* mRNA expression with the prognosis of IS cases in the follow-up study

Outcome	Events	Person-years	Incidence density (/10^4 Person-years)	*HR* (95% *CI*)	*HR* (95% *CI*) [a]
All cause death	28	580.595	482.264	0.981 (0.885-1.087) *P*=0.711	0.962 (0.842-1.099) *P*=0.571
Stroke death	16	570.301	280.554	0.992 (0.951-1.036) *P*=0.731	0.986 (0.908-1.071) *P*=0.739
Ischemic stroke death	13	567.375	229.125	0.986 (0.888-1.095) *P*=0.789	0.975 (0.821-1.158) *P*=0.774

[a] *Adjusted for age, gender, smoking, drinking, hypertension, diabetes and dyslipidemia.*
