# Supplementary figures and images for: Association of THBS1 genetic variants and mRNA expression with the risks of ischemic stroke and long-term death after stroke
Source: Front Aging Neurosci. 2022 Sep 23;14:1006473. doi: 10.3389/fnagi.2022.1006473 (PMC9545898; doi:10.3389/fnagi.2022.1006473)

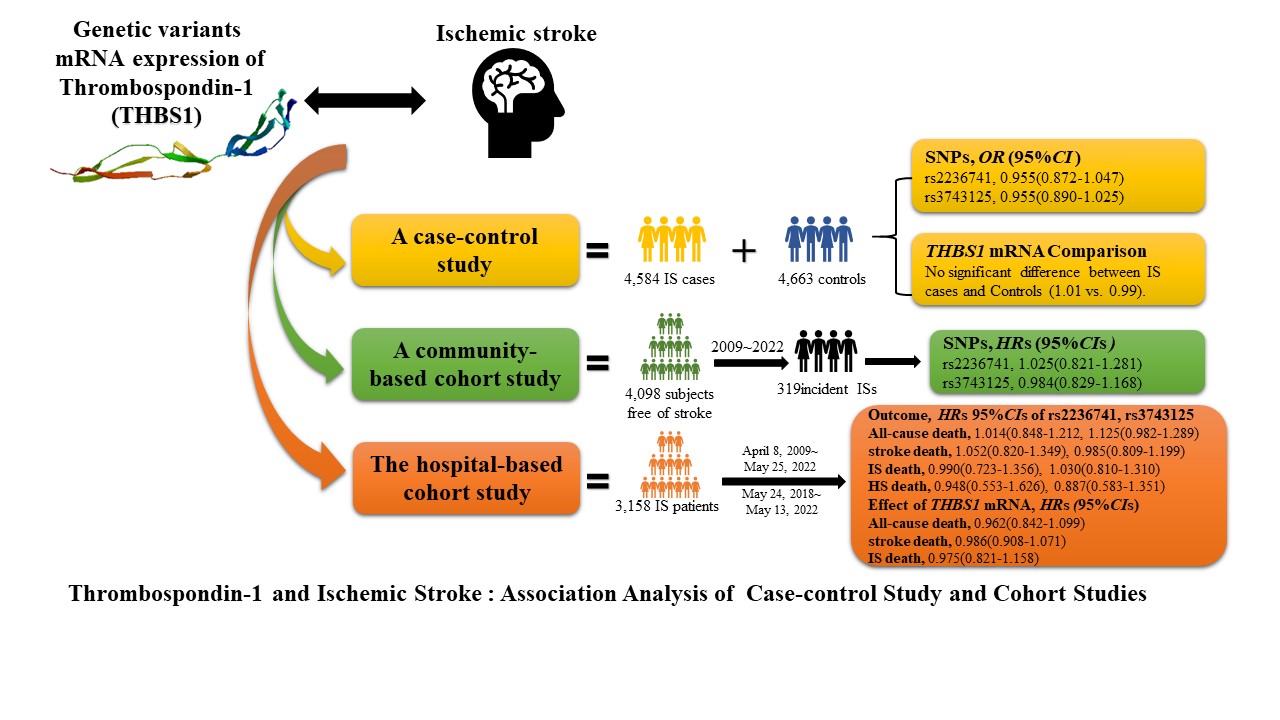

Supplement: Supplementary file 2 [file Image_1.JPEG]
